# Mapping altered brain connectivity and its clinical associations in adult moyamoya disease: A resting-state functional MRI study

**DOI:** 10.1371/journal.pone.0182759

**Published:** 2017-08-04

**Authors:** Ken Kazumata, Khin Khin Tha, Haruto Uchino, Masaki Ito, Naoki Nakayama, Takeo Abumiya

**Affiliations:** 1 Department of Neurosurgery, Hokkaido University Graduate School of Medicine, Kita, Sapporo, Japan; 2 Department of Radiation Medicine, Hokkaido University Graduate School of Medicine, Kita, Sapporo, Japan; University of Texas at Austin, UNITED STATES

## Abstract

Detection of subtle ischemic injuries in moyamoya disease may enable optimization of timing of revascularization surgery, and could potentially improve functional outcomes. Resting-state functional magnetic resonance imaging (rs-fMRI) is widely used to study functional organization of the brain, but it remains unclear whether rs-fMRI could elucidate distinct characteristics in moyamoya disease. Here, we aimed to determine changes in a conventional rs-fMRI measure and analyze any associations with clinical symptoms and cerebral hemodynamics. Thirty-one adults with moyamoya disease and 25 adult controls underwent rs-fMRI, in which we measured brain connectivity via temporal correlations of low-frequency BOLD signals. We identified the extent of between-group differences with multivoxel pattern analysis. Seed-based analysis was performed to determine associations with vascular lesions, symptoms, and regional cerebral blood flow (rCBF). There was significantly altered connectivity in the precentral gyrus, operculo-insular region, precuneus, cingulate cortex, and middle frontal gyrus in moyamoya disease. There was reduced connectivity in the left insula, left precuneus, right precentral, and right middle frontal regions, which form part of the salience, default mode, motor, and central executive networks, respectively. Patients with ischemic motor-related symptoms showed significantly decreased connectivity in precentral homotopic regions compared with those without, while there were no differences in vascular lesions or rCBF. Connectivity between the right occipital and left hippocampus was significantly associated with cognitive performance and posterior cerebral artery involvement. Our results demonstrate distinct alterations in the temporal correlations of low-frequency BOLD signals, predominantly in resting-state networks in moyamoya disease. Additionally, rs-fMRI measures were associated with ischemic motor-related symptoms and cognitive performance in the patients. Thus, rs-fMRI may offer a useful non-invasive method of acquiring additional information beyond cerebral perfusion as part of clinical investigations in patients with moyamoya disease.

## Introduction

Resting-state functional magnetic resonance imaging (rs-fMRI) is a recently validated tool for assessing brain function that measures spontaneous brain activity via low-frequency blood-oxygen-level-dependent (BOLD) signals. rs-fMRI can reveal associations between neuroanatomy, fluid intelligence, attention, and task-induced brain activity [1–8]. rs-fMRI allows investigation of functional connectivity of distant brain regions without requiring task performance in patients with neurological deficits [9,10]. This technique has more recently been applied in the study of neurobehavioral dysfunction in stroke patients, as well as vascular cognitive impairments [9,11]. rs-fMRI assesses functional organization of the intact brain in patients who have experienced cerebral infarction, providing predictors of functional outcomes and markers to evaluate therapeutic interventions [9,12].

Moyamoya disease (MMD) is a rare cerebrovascular disease characterized by a fine vascular network (“moyamoya vessels”) at the base of the brain, which results from stenosis or occlusion of the terminal branches of the internal carotid arteries [13]. In addition to cerebral stroke, patients with this condition may present with cognitive impairments, such as executive dysfunction, impaired working memory, and attention deficit, likely as a result of chronic ischemic injury [14–16]. Early detection of altered brain function due to ischemia could potentially improve the quality of life of such patients, in whom chronic ischemia can lead to suboptimal brain development in children and adolescents, and/or the early onset of cognitive decline in adults [14–18].

Temporal correlations in low-frequency (0.008–0.09 Hz) blood-oxygen-level-dependent (BOLD) signals are widely used as markers of spontaneous brain activity in rs-fMRI. However, previous research has demonstrated the critical contribution of perfusion delay to BOLD signals in MMD, suggesting that alterations in functional connectivity stem from perfusion abnormalities rather than having neuronal origins [19]. Nevertheless, recent studies have encouraged the application of rs-fMRI to investigate brain function in MMD. Such research has revealed several patterns of brain function associated with MMD. For example, the averaged square root of power maps of low-frequency BOLD signals was found to identify disease-related changes in patients with MMD and predict postsurgical cognitive improvement [17,20]. Furthermore, attenuated local synchronization of low-frequency BOLD signals has been associated with worse cognitive performance in patients with MMD [17]. Although conventional rs-fMRI analysis is performed by using the temporal correlations of low-frequency BOLD signals, profiles of this parameter have not been sufficiently reported in patients with MMD. In particular, it has not been determined whether the temporal correlations of low-frequency BOLD signals are altered in patients with an otherwise apparently healthy brain, who are potential candidates requiring early detection of cognitive impairments. Previous investigations using voxel-based analysis have demonstrated altered distribution of regional cerebral blood flow (rCBF) in MMD, before and after the revascularization surgery [21,22]. However, the extent of alterations in low-frequency BOLD signals associated with vascular lesions and rCBF has not been clearly demonstrated. Furthermore, the clinical significance of such alterations has not been determined in relation to ischemic symptoms or cognitive performance [17,23]. Thus, elucidating pattern of connectivity measured with rs-fMRI is worthwhile because this novel technique offers a potentially feasible method to assess multiple brain systems and could provide additional information beyond cerebral perfusion as part of routine clinical investigations.

In this study, we investigated altered connectivity associated with MMD by measuring temporal correlations of low-frequency BOLD signal with rs-fMRI. Specifically, we determined regional and whole-brain spatial characteristics of these alterations, and quantified potential associations with ischemic symptoms, severity of vascular involvements, regional cerebral blood flow, and cognitive performance.

## Materials and methods

### Participants

This prospective study was approved by the research ethics committee of Hokkaido University Hospital. All study participants provided written informed consent.

The inclusion criteria were a clinical diagnosis of idiopathic MMD based on the consensus criteria and guidelines for MMD proposed by the Research Committee on Spontaneous Occlusion of the Circle of Willis, and being aged over 20 years [24]. We excluded patients with cortical infarctions larger than 3 cm, a previous history of intracranial hemorrhage, or previous revascularization surgery. After exclusions, 31 patients (9 men, 22 women; age range, 21–60 years; mean age, 41.9 ± 10.8 years) were enrolled for image analysis. Patients were selected over a period of 33 months (from June 2013 to April 2016).

The inclusion criteria for controls were no clinical evidence of psychiatric or neurological disorders, normal intelligence quotient (IQ), no brain lesions on conventional MRI, and no medication that could affect cognitive function. The control group comprised 25 subjects (9 men, 16 women; age range, 27–56 years; mean age, 38.8 ± 8.1 years).

### Neuropsychological assessment

A normal IQ was confirmed in control subjects with the Japanese version of the Nelson Adult Reading Test [25]. In the patient group, a full-scale IQ score, verbal and performance IQ scores, verbal comprehension, perceptual organization (PO), working memory (WM), and processing speed (PS) were assessed with the Wechsler Adult Intelligent scale-III. Four patients were not able to complete the neuropsychological examination, and were excluded from the correlation analysis exploring associations with cognitive performance.

### Neuroimaging data

#### Evaluation of cerebral perfusion

rCBF was evaluated in the patients with either [^123^I] N-isopropyl p-iodoamphetamine (IMP) (n = 27), 99m Tc-propylene-amine oxime (PAO) (n = 1) in single-photon computed tomography (SPECT), or O-15 (n = 3) gas with positron-emission tomography (PET). Of the patients in which rCBF was measured with IMP/SPECT, 25 were assessed with a SPECT scanner (GCA-9300R; Toshiba Medical Systems Corporation Ltd, Tochigi, Japan) at Hokkaido University. The other 2 patients were imaged with [123I]-IMP/SPECT at other hospitals with different acquisition modalities. Accordingly, we restricted the post-hoc quantitative analysis to the former 25 patients.

Patients underwent SPECT/PET and rs-fMRI evaluation within 7 days of admission as part of routine clinical assessments to evaluate surgical indication. After a 1-min intravenous bolus infusion of 167 MBq of 123I-IMP (5 mL) with physiologic saline flush infusion (5–10 mL), data were acquired 20 min after the injection with a total scan duration of 20 min. SPECT images were spatially normalized to the tissue probability map available in Statistical Parametric Mapping version 12 (SPM12, Wellcome Department of Cognitive Neurology, University College London, London, UK. www.fil.ion.ucl.ac.uk/spm/). Region of interest (ROI) analysis was performed to extract rCBF values from the images in standardized space using automated anatomical labeling (AAL) [24]. To eliminate inter-scan variability, we globally normalized voxel values with proportional scaling to a mean voxel value of 50 ml/100 mL/min.

#### Magnetic resonance image acquisition

MRI was performed with a 3 T scanner (Achieva TX; Philips Medical Systems, Best, the Netherlands). A standard 32-channel RF head coil (Philips Medical Systems, Best, Netherlands) was used for signal reception. Three-dimensional magnetization-prepared rapid acquisition gradient echo (3D-MPRAGE) was performed with a repetition time (TR), echo time (TE), and flip angle of 6.8 ms, 3.1 ms, and 8°, respectively, and an inversion time (TI) of 1100 ms. During the rs-fMRI experiment, all participants were asked to relax, move as little as possible, keep their eyes open, and try not to think about anything specific. rs-fMRI was performed with the following parameters: TR, 3000 ms; TE, 30 ms; flip angle, 80°; slice thickness, 3.3 m; slice spacing, 3.3 mm; number of volumes, 140; and number of slices, 48; total scan time, 7 min 9 sec.

In addition to the above imaging sequences, we also acquired axial fast spin-echo T2-weighted (T2WI) images (with TR, 5059 ms; TE, 90 ms; and echo train length (ETL), 15), axial fast fluid-attenuated inversion recovery (FLAIR) images (with TR, 10000 ms; TE, 100 ms; and TI, 2700 ms), and 3D-time-of-flight magnetic resonance angiograms (3D-TOF-MRA) (with TR, 20 ms; TE, 3.5 ms; and flip angle, 18).

#### Evaluation of vascular lesions

Severity of occlusive lesions in the middle cerebral artery (MCA), anterior cerebral artery, and posterior cerebral artery (PCA) were assessed with MRA. We classified lesions into 3 grades, as follows: 0, normal/mild stenosis; 1, moderate stenosis (majority of distal branches are visible); 2, severe stenosis/occlusion (majority of distal branches are not visible) (Table 1). In the post-hoc analysis, we compared temporal correlations of low-frequency BOLD signals between patients with unilateral disease and bilateral disease. We defined unilateral disease where arterial lesions of the MCA of either hemisphere were rated as 0, while MRA score of MCA in the other hemisphere demonstrated either grade 1 or 2.

**Table 1:** Frequency (%) and severity (MRA score) of vascular involvement evaluated on magnetic resonance angiography (MRA).

MRA score	Rt. MCA	Lt. MCA	Rt. ACA	Lt. ACA	Rt. PCA	Lt. PCA
0	12.9	45.2	45.2	41.9	87.1	87.1
1	16.1	22.6	22.6	22.6	6.5	3.2
2	71.0	32.3	32.3	35.5	6.5	9.7

Rt: right, lt: left, MCA: middle cerebral artery, ACA: anterior cerebral artery, PCA: posterior cerebral artery. For the MRA score, three grades were classified based on MRA data as follows: 0, normal/mild stenosis; 1, moderate stenosis (majority of distal branches are visible); 2, severe stenosis/occlusion (majority of distal branches are not visible).

#### rs-fMRI image preprocessing

Preprocessing of rs-fMRI data was performed with SPM12 (http://www.fil.ion.ucl.ac.uk/spm/), implemented in MATLAB (Matlab 8.6.0, Release 2015b, Mathworks Inc., Sherborn, MA, USA). We performed temporal preprocessing with the functional connectivity toolbox (Conn version 16, http://www.alfnie.com/software) (Supplementary information) [26]. The first 10 volumes of rs-fMRI scans were discarded to allow for magnetic field stabilization. Slice-timing correction was performed to align rs-fMRI images to the center of the image. Each image volume was then realigned to the first volume to correct for any residual head movement.

Individual 3D-MPRAGE structural images were segmented into grey matter, white matter, and cerebrospinal fluid (CSF) with the Diffeomorphic Anatomical Registration Through Exponentiated Lie (DARTEL) algorithm [27]. Structural images were co-registered to the first volume of functional images after realignment, using a 6 degrees-of-freedom linear transformation without re-sampling. Co-registered structural images were standardized to the Asian brain template defined by the Montreal Neurological Institute (MNI), and a transformation matrix was applied to the corresponding functional, gray matter, white matter, and CSF images. Spatial smoothing was then applied with the full width at half-maximum equal to 6 mm.

The head movement time-series, white matter signal, and CSF signal were regressed out from each voxel using a CompCor strategy [28]. Temporal smoothing for the time course was performed with a band-pass filter (0.008–0.09 Hz). The CONN toolbox was used to perform voxel-by-voxel and seed-based analyses by computing the temporal correlations between the BOLD signals from a given voxel (or ROI) with all other voxels (or ROIs) in the brain. ROIs were placed using the automated anatomic (AAL) atlas available in the Conn plug-in. Pearson correlations for all time-course pairs were computed for each participant and transformed into z-scores via Fisher's transformation.

To create maps of resting-state networks, we applied FSL Multivariate Exploratory Linear Optimized Decomposition into Independent Components (MELODIC) for independent component analysis (ICA) (FMRIB, University of Oxford, UK; www.fmrib.ox.ac.uk/fsl/melodic2/index.html). For each participant, the smoothed and normalized functional images were concatenated across time in a single 4D image. The MELODIC algorithm uses probabilistic ICA to estimate the number of relevant noise and signal sources in the 4D data. Probabilistic ICA provides intensity values (z scores) and thus a measure of the contribution of the time course of a component to the signal at a given voxel. We chose to set the ICA analysis to deliver 30 components. Resting-state networks were identified with reference to previously published templates available online (http://www.brainnexus.com/resources/resting-state-fmri-templates).

### Data analysis

Connectivity of the brain was analyzed by the following steps. Firstly, we used multivariate pattern analysis (MVPA) implemented in the connectome-MVPA CONN toolbox to elucidate the extent of differences between patients and controls subjects, as well as to identify seed regions for further post-hoc analysis [26]. Analysis using MVPA has been described in elsewhere [29]. Briefly, MVPA assesses the multivariate pattern of connections between each voxel and every other voxel in the brain by performing a principal component analysis, yielding an unbiased mapping of brain areas showing abnormal whole brain connectivity patterns. In the present study, each voxel was assumed to have a 3-dimensional representation of the spatial pattern of its connectivity to all other voxels within each participant. Subsequently, second-level analyses were performed to test for group differences in whole-brain connectivity by means of an F-test performed across all connectivity patterns of all voxels in 3D space. Statistical parametrical connectivity maps were thresholded at the voxel level of p < 0.001, uncorrected (two tailed), and at the cluster level of p < 0.05, family-wise error (FWE) corrected. Secondly, to determine the profiles altered brain connectivity, we performed seed-to-voxel mapping in selected regions identified in a group comparison using MVPA. The threshold for significance was set at p < 0.05 and results of the exploratory seed-to-voxel analyses were considered significant if clusters survived FWE correction at p < 0.05.

We also investigated whether significant changes in rs-fMRI parameters were associated with the severity of vascular lesions, ischemic symptoms, and neuropsychological examination scores. Following the seed-to-voxel analysis in selected regions after MVPA, the values of temporal correlation of low-frequency BOLD signals were extracted using ROIs defined with the AAL, which included clusters with the most significant changes in each selected region.

Correlations with cognitive test performance were explored within the patient group. ROI-to-ROI connections were explored using a general linear model, implemented in the Conn second-level analysis. Correlations with cognitive performance were examined using subtest scores of the WAIS–III (WM, PO, and PS), which were included as separate covariates in the model. The effect of subtest scores for PO, WM, and PS on ROI-to-ROI connections were explored by applying a threshold of p < 0.001 (uncorrected) and p < 0.05 (FDR corrected). Associations between cognitive performance and temporal correlations of low-frequency BOLD signals were also quantified using Pearson product-moment correlation coefficients.

## Results

### Characteristics of study participants

The mean estimated IQ of controls was 106.0 ± 7.7 (range, 94–116). The mean full scale IQ of patients was 92.0 ± 18.1 (range, 54–121), such that 29.0% of patients were below < 1 SD (i.e., fIQ < 85). Performance IQ of patients was lower than verbal IQ (verbal vs. performance, 94.1 ± 18.7 vs. 91.0 ± 15. 5, p < 0.05, one-tailed *t*-test). Among patients, clinical presentation varied, with some patients presenting as asymptomatic (n = 18), while others presented with transient ischemic attack (TIA; n = 12) and minor stroke (n = 1). Conventional MRI revealed white matter high intensity on T2/FLAIR in 9 patients, and small cortical infarctions of less than 3 cm in 2 patients. There was a trend to greater involvement of right side MCA compared with the left (p = 0.09). The average full scale IQ was 92.9±16.2 in asymptomatic patients and 94.2±18.5 in patients with TIA. The full scale IQ in patients with minor stroke was 54.

### Characteristics of temporal correlations of low-frequency BOLD signals

**Fig 1:**
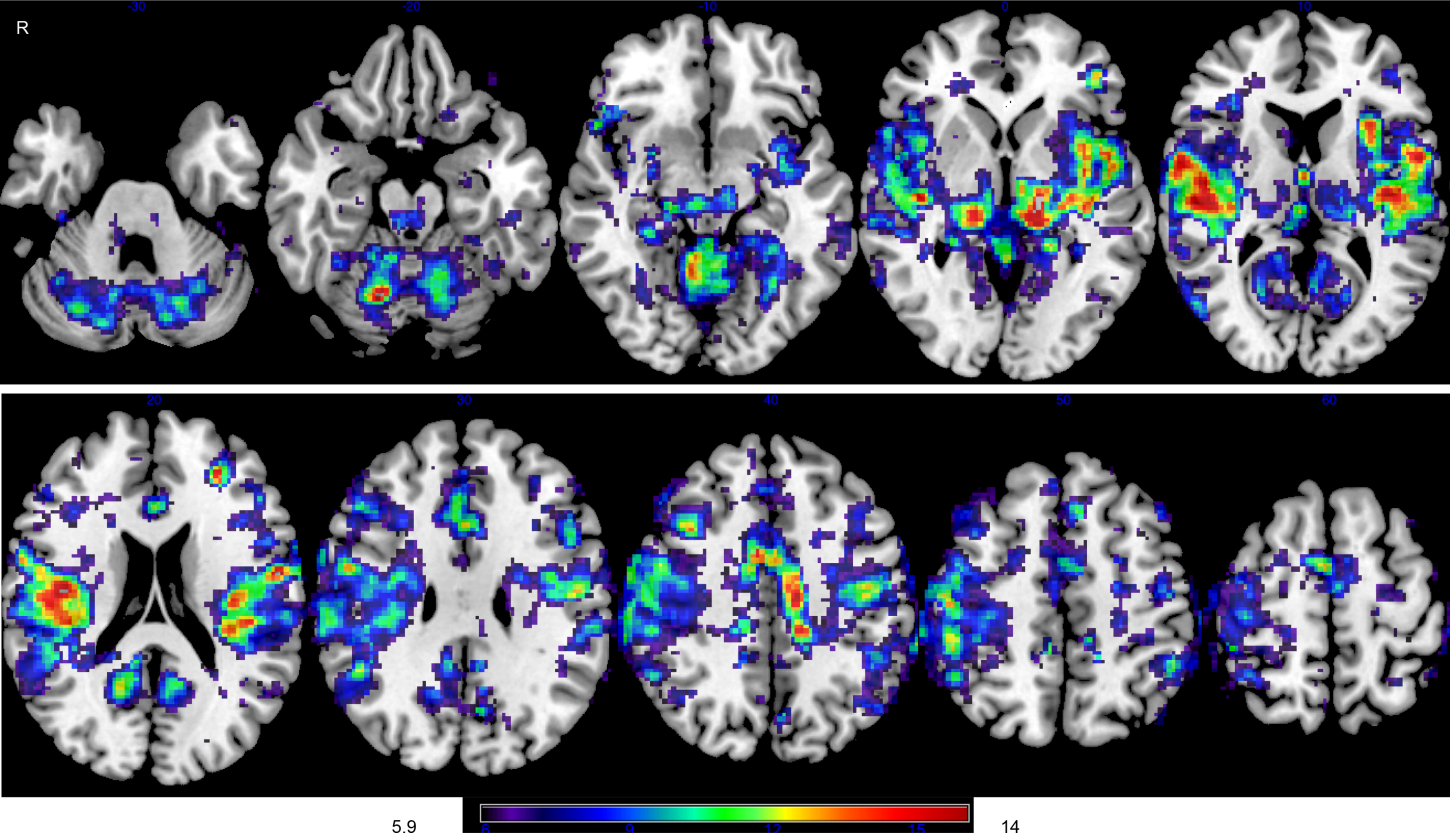
Voxel-level analysis of altered temporal correlations of low-frequency blood-oxygen-level-dependent (BOLD) signals in moyamoya disease (MMD). Brain regions in which there are significant differences in temporal correlations of low-frequency BOLD signals between patients with MMD and controls were explored at voxel level with multivariate pattern analysis (MVPA). The color bar indicates the F-statistic of between-group differences in terms of the spatial maps of the three first principal components.

**Fig 2:**
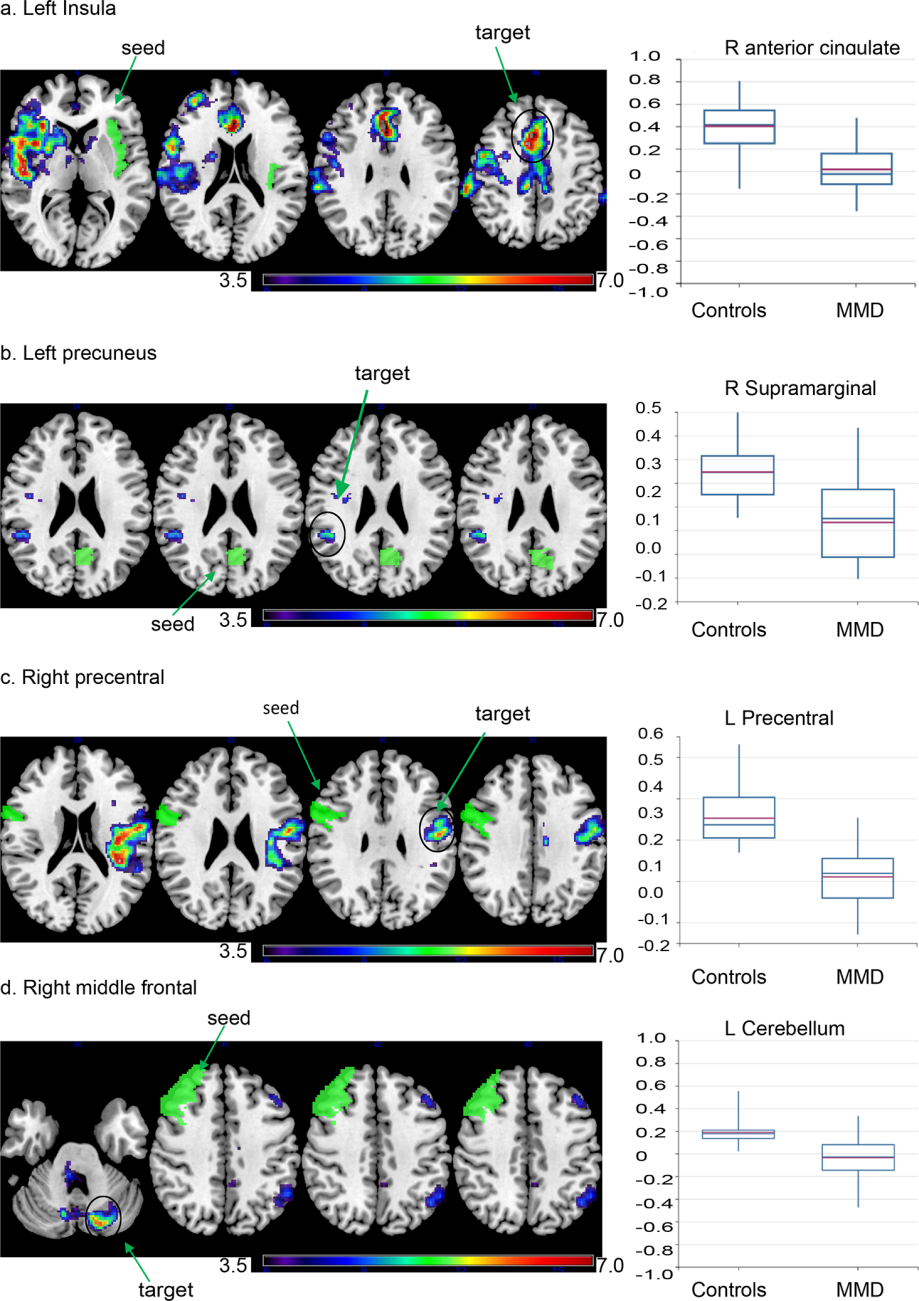
Decreased temporal correlations of low-frequency BOLD signals are present within regions comprising resting-state networks. Seed regions were selected based on a whole-brain voxel-based analysis and are depicted in green. Voxels that display significant changes in connectivity with the left insula (a), left precuneus (b), right precentral (c), and right middle frontal gyrus (d) are demonstrated (explorative voxel-level threshold at p < 0.001 and FWE-corrected at cluster level p < 0.05). The color bar indicates the Fisher-transformed Z score of between-group differences. Box plots indicate the Fisher-transformed Z score of each group in clusters showing the greatest changes. Resting state networks corresponding to each seed region are demonstrated as a reference. Maps represents salience, default mode, motor, and executive networks generated through independent component analysis in healthy controls.

The MVPA showed group differences in whole-brain temporal correlations of low-frequency BOLD signals (Fig 1). The largest cluster in which there was a significant group difference between patients and controls included the right pre- and postcentral gyri, bilateral precuneus, the left precentral gyrus, the right middle frontal gyrus, bilateral anterior cingulate gyrus, and the left insular cortex. Seed-to-voxel analyses were subsequently performed to investigate the characteristics of altered temporal correlations of low-frequency BOLD signals in each region. We defined ROIs in the left insula, left precuneus, right precentral gyrus, right anterior cingulate gyrus, and right middle frontal gyrus based on the results of the MVPA analysis. The left insula showed decreased correlations in the bilateral anterior to middle segment of the cingulate gyrus, as well as in the right operculo–insular cortex (Fig 2). There were reduced temporal correlations of low-frequency BOLD signals between the right anterior cingulate gyrus and bilateral operculo–insular regions. There were also reduced temporal correlations of low-frequency BOLD signals between the left precuneus and the right supramarginal gyrus (Fig 2), while the right precentral gyrus showed decreased correlations with the left precentral gyrus (Fig 2). The right middle frontal gyrus showed decreased correlations with the left inferior parietal lobe, middle frontal gyrus, and left cerebellar hemisphere (Fig 2).

The right precentral gyrus (2390 voxels), right postcentral gyrus (2058 voxels), precuneus (1770 voxels), left precentral gyrus (1528 voxels), right middle frontal gyrus (1327 voxels), anterior segment of the cingulate gyrus (1143 voxels), and left insular cortex (110 voxels) are present within the largest cluster of significant difference. In the F-maps, a primary threshold of p < 0.001 uncorrected was applied and clusters of connections were thresholded at p < 0.05, family-wise error (FWE) corrected.

Target locations showing the highest correlations are indicated with Montreal Neurological Institute (MNI) coordinates (x, y, z) (mm) for each seed. The patients with MMD show reduced connectivity between the left insula and the anterior segment of the cingulate gyrus (+2, +24, +24), right insular cortex, opercular regions, and precentral gyrus (a). Furthermore, patients with MMD show reduced connectivity between the left precuneus and right supramarginal gyrus (+46, -42, +26) (b). Moreover, patients with MMD show reduced connectivity between the right precentral gyrus and left precentral gyrus (-48, -08, -02) (c). Patients with MMD also show reduced connectivity between the right middle frontal gyrus and left middle frontal gyrus, left inferior parietal lobe, and left cerebellum (-18, -78, -28) (d).

### Correlations with cognitive performance

**Fig 3:**
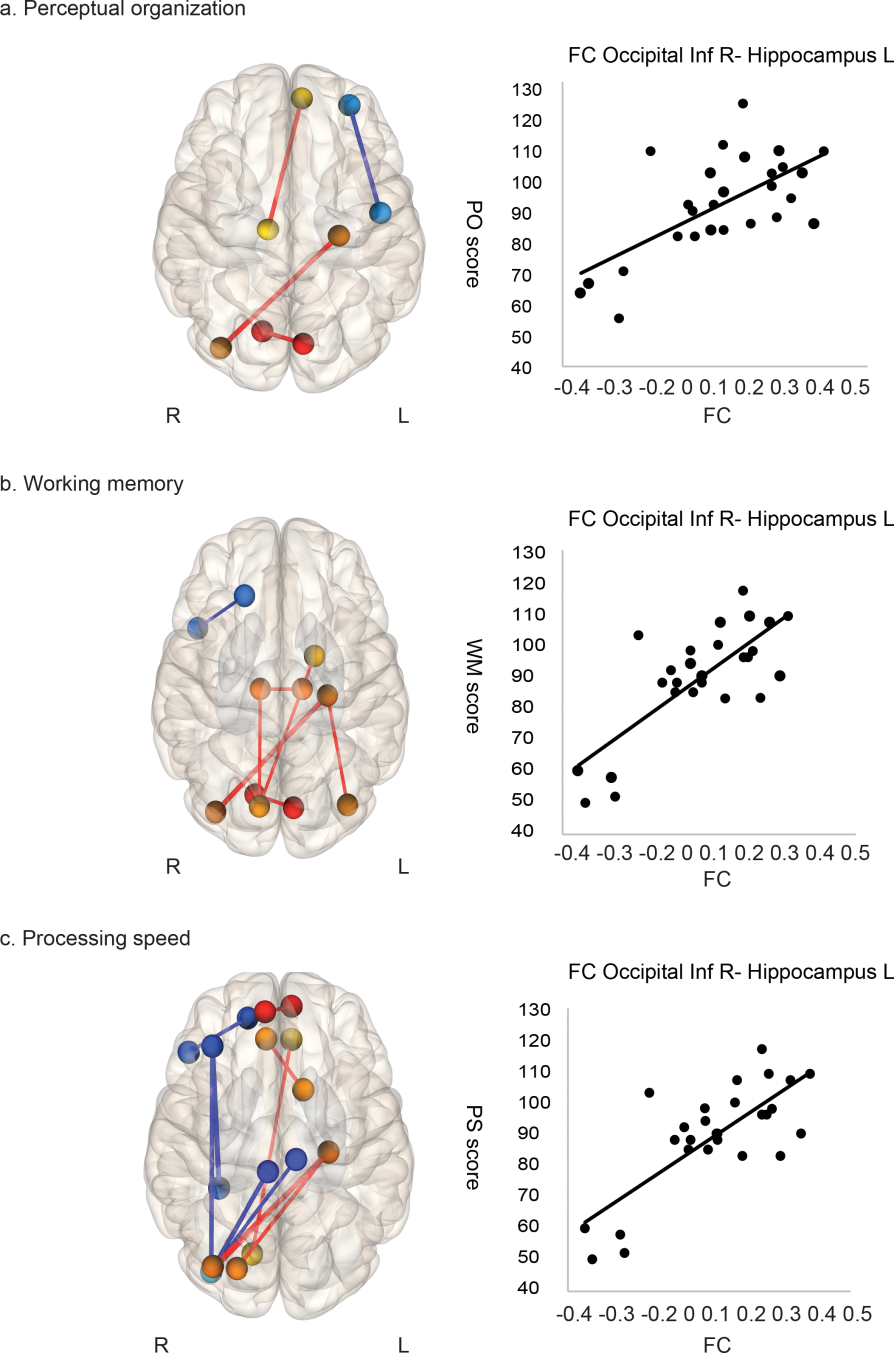
Temporal correlations of low-frequency blood-oxygen-level-dependent (BOLD) signals are associated with cognitive performance in moyamoya disease. Associations of temporal correlations of low-frequency BOLD signals with perceptual organization (a), working memory (b) and processing speed (c) were explored, and significant associations were overlaid on a 3D template in MNI space (p < 0.001, uncorrected). The circles show brain areas that have statistically significant functional connections. The color bar indicates t statistics. A. Perceptual organization (PO) scores are associated with connections between the visual area, visual association area, and prefrontal association area. The scatter plot shows the representative correlations between connectivity of the left hippocampus/right inferior occipital region and PO scores (r = 0. 65, p < 0.001). b.Working memory (WM) is associated with multiple connections among the visual area, visual association area, and prefrontal association area. The scatter plot shows representative correlations between connectivity of the left hippocampus/right inferior occipital region and PS scores (r = 0. 76, p < 0.001). c. Processing speed (PS) is associated with multiple connections between the visual area, visual association area, and prefrontal association area. The scatter plot shows representative correlations between connectivity of the left hippocampus/right inferior occipital region and PS scores (r = 0. 72, p < 0.001).

Fig 3 and Table 2 show the associations between ROI-to-ROI connectivity and neuropsychological test scores. In patients with MMD, there was a positive correlation between PO, WM, and PS scores and ROI-to-ROI connections. The strongest correlation was found in the connection between the left hippocampus and right inferior occipital lobe (PO, p = 0.02; WM, p = 0.0004; PS, p = 0.002, FDR-corrected). Furthermore, there was a negative correlation between PS scores and connectivity between the right middle frontal and right inferior occipital lobes (p = 0.003, FDR-corrected).

**Table 2:** Connections showing significant correlation with cognitive performance.

Connections	T value	p-uncorrected	p-FDR
**Perceptual organization**			
Hippocampus L- Inferior occipital R	4.28	0.0002	0.0212
**Working memory**			
Hippocampus L-Inferior occipital R	5.86	0	0.0004
Calcarine R-Cuneus L	4.21	0.0003	0.0259
Hippocampus L-Inferior occipital L	4.02	0.0005	0.0211
Thalamus R-Thalamus L	3.9	0.0006	0.0305
Cuneus R-Thalamus R	3.87	0.0007	0.0258
Cuneus R-Pallidum L	3.81	0.0008	0.0258
Cuneus R-Thalamus L	3.78	0.0009	0.0258
Anterior cingulate gyrus R-Olfactory L	3.72	0.001	0.0452
**Processing speed**			
Inferior occipital R-Hippocampus L	5.19	0	0.0018
Middle occipital R-Hippocampus L	4.2	0.0003	0.0263
Hippocampus L-Superior occipital R	4.06	0.0004	0.0127
Inferior occipital R-Paracentral lobule L	-3.96	0.0005	0.0121
Triangular part of inferior frontal gyrus R-Superior orbitofrontal gyrus R	-4.14	0.0003	0.0311
Middle frontal gyrus R-Inferior occipital R	-4.82	0.0001	0.0027
Inferior occipital R-Paracentral lobule R	-4.91	0	0.0018
Middle frontal R-Fusiform R	-5.38	0	0.0013

R; right, L; left

### Post-hoc analysis

We considered that connectivity between the right and the left precentral gyrus (primary motor cortex, M1) [3,30] was a candidate for post-hoc analysis to investigate associations with ischemic motor-related symptoms, vascular lesions in the MCA, and rCBF. There was a significant between-group difference in the temporal correlations of low-frequency BOLD signals (ischemic motor-related symptoms (+), n = 12 vs. no ischemic motor-related symptoms, n = 19; p = 0.012). However, no significant between-group difference was observed in the severity of vascular lesions assessed with a composite MRA score for the MCA, ACA, and PCA. Furthermore, there was no significant difference in rCBF of the precentral gyrus between the two groups. Unilateral MMD showed a trend to reduced temporal correlations of low-frequency BOLD signals compared with bilateral MMD (p = 0.068). No significant correlation was found between asymmetry of rCBF and the temporal correlations of low-frequency BOLD signals connectivity of the precentral homotopic region.

We also investigated the contribution of PCA involvement, since significant correlations with cognitive performance were observed in ROIs distributed throughout the PCA. Patients with PCA involvement showed significant decreases in temporal correlations of low-frequency BOLD signals between the left hippocampus and right inferior occipital lobe compared with patients without PCA involvement (p = 0.038).

## Discussion

Cognitive dysfunction resulting from MMD has begun to garner the attention of researchers studying the disorder [14,15,18]. However, despite the increasing use of rs-fMRI to investigate brain function in other cognitive disorders, few published studies have used rs-fMRI to investigate cognitive dysfunction in MMD, [17,20,23]. In this study, we conducted rs-fMRI in a group of patients with MMD who had minimal ischemic lesions and no prior history of revascularization surgery, because such patients require early detection of ischemic brain injury. Here, we used a conventional measure, temporal correlations of low-frequency BOLD signals, to evaluate functional connectivity. We found significant changes in temporal correlations of low-frequency BOLD signals in patients predominantly in areas that represent resting-state networks. We also found associations between temporal correlations of low-frequency BOLD signals of the motor network and ischemic motor-related symptoms. Moreover, even though cognitive dysfunction in MMD has been attributed to frontal lobe dysfunction, cognitive impairments were predominantly associated with connectivity in regions located within the PCA. Although perfusion abnormalities in MMD prevent interpretation of the results showing altered brain activity, we suggest that rs-fMRI enables assessment of multiple brain system as part of routine clinical investigations.

In our sample, rs-fMRI demonstrated distinct changes in temporal correlations of low-frequency BOLD signals, i.e., functional connectivity, predominantly in the precentral gyrus, operculo–insular region, cingulate cortex, middle frontal lobe, and precuneus. The identified regions correspond to core structures within resting-state networks, including salience, default mode, motor, and fronto-parietal networks [1,31,32]. Resting-state networks are known to be robust when both cerebral perfusion and metabolism remain normal [1,33]. The present study illustrates that vascular lesions in MMD impair resting-state networks. Similar findings of aberrations in resting-state networks in MMD have been demonstrated with other rs-fMRI parameters, such as regional homogeneity [17]. Interestingly, the spatial pattern in the present study resembled the time-lag map of low-frequency oscillations (LFOs) described in a previous study [34]. LFOs are hypothesized to have a systemic origin, which is propagated within the blood stream, beginning near the pre- and post-central gyri, the orbitofrontal gyrus, and the posterior cingulate cortex [34,35]. In MMD, the arrival of low-frequency BOLD signals can delay this significantly [23]. Thus, it is speculated that perfusion abnormalities in MMD would affect perfusion-metabolic coupling predominantly in areas with faster arrival of BOLD signals, which would therefore demonstrate selective disruption of resting-state networks.

Previous studies suggest that rs-fMRI can be used to evaluate hemodynamic insufficiency. In the present study, while severity of vascular lesions and rCBF both failed to show distinct differences between patients with and without ischemic motor-related symptoms, a significant decrease was observed in the connectivity of M1 in patients with motor-related symptoms compared with those without such symptoms. This finding is consistent with previous studies where alterations of functional connectivity in the homotopic M1 during rest were correlated with motor function during recovery from stroke [10,36]. Although MMD likely alters functional connectivity measures via non-neuronal mechanism, the association with WAIS-III index scores suggests that rs-fMRI does reflect brain function even in MMD. The present study suggests that altered connectivity in the PCA can serve as a marker for cognitive dysfunction. Previous clinical investigations have also reported that PCA involvement is associated with cognitive dysfunction, supporting our results [37]. Furthermore, cognitive performance measured with PO is spatially associated with the right parietal, occipito-parietal, and superior temporal cortices in healthy individuals, which may further explain the spatial characteristics of our results [38]. Although frontal lobe ischemia has been considered as the main mechanism leading to cognitive impairments in MMD, the present study provides supportive evidence of an additional contribution of the PCA. Negative correlations between PS and right prefrontal association areas (middle frontal gyrus, inferior frontal gyrus, and paracentral lobe), as well as between the right prefrontal association areas and occipital region were also observed. Altered cerebral hemodynamics in prefrontal association areas could inadvertently increase synchronization of perfusion dynamics with other brain regions with no particular functional associations.

This study had some limitations worthy of mention. First, a small number of patients were investigated. Despite the statistical significance of our findings, the sample size should not be considered sufficient to allow generalization of the results to entire populations. The association with cognitive impairment should also be interpreted as preliminary. Second, although the present study did not reveal associations between temporal correlations of low-frequency BOLD signals and rCBF, either a delay or the amount of perfusion through the small arteries could be associated with temporal correlations of low-frequency BOLD signals. rCBF measured with SPECT reflects tissue perfusion, which is not always equivalent to the perfusion through the small cortical arteries because of the development of collateral circulation [39]. The contribution of cerebral perfusion should be assessed within the time frame of rs-fMRI acquisition [33,40]. In particular, the time delay discussed above could have contributed markedly to the strength of the measured functional connectivity in MMD. Indeed, task-free fMRI has been applied to measure the time delay, as has cerebrovascular reactivity, and is a promising approach for investigating cerebral perfusion in MMD [41]. Third, seed-based analysis was employed in this study. An alternative method, such as ICA, could yield disease-related networks from rs-fMRI data without requiring the placement of predefined ROIs [1,42]. Nonetheless, previous investigations have employed univariate voxel-wise group level comparisons to characterize local changes within a specific component map [43,44]. MVPA identifies principal component imaging features within the observed temporal fluctuations. Thus, we consider that the seed-based analysis was optimal for investigating region-level changes.

The present study demonstrated distinct alterations in temporal correlations of low-frequency BOLD signals using rs-fMRI in patients with MMD. These alterations were observed predominantly in resting-state networks. Additionally, rs-fMRI measures were associated with ischemic motor-related symptoms and cognitive performance in MMD. Thus, considering its noninvasiveness, rs-fMRI may be a useful clinical tool to acquire additional information beyond cerebral perfusion as part of routine clinical investigations in patients with MMD.
